# Genome-Wide Mapping of Susceptibility to Coronary Artery Disease Identifies a Novel Replicated Locus on Chromosome 17

**DOI:** 10.1371/journal.pgen.0020072

**Published:** 2006-05-19

**Authors:** Martin Farrall, Fiona R Green, John F Peden, Per G Olsson, Robert Clarke, Mai-Lis Hellenius, Stephan Rust, Jacob Lagercrantz, Maria Grazia Franzosi, Helmut Schulte, Alisoun Carey, Gunnar Olsson, Gerd Assmann, Gianni Tognoni, Rory Collins, Anders Hamsten, Hugh Watkins

**Affiliations:** 1 Department of Cardiovascular Medicine, University of Oxford, Oxford, United Kingdom; 2 School of Biomedical and Molecular Sciences, University of Surrey, Guildford, United Kingdom; 3 AstraZeneca AB, Mölndal, Sweden; 4 Clinical Trials Service Unit and Epidemiological Studies Unit, University of Oxford, Oxford, United Kingdom; 5 Department of Medicine, Karolinska Institute, Stockholm, Sweden; 6 Leibniz—Institut für Arterioskleroseforschung an der Universität Münster, Münster, Germany; 7 Department of Cardiovascular Research, Istituto di Ricerche Farmacologiche Mario Negri, Milano, Italy; 8 Oxagen Limited, Abingdon, Oxfordshire, United Kingdom; 9 Institute for Clinical Chemistry and Laboratory Medicine, University Clinics Münster, Münster, Germany; University of Michigan, United States of America

## Abstract

Coronary artery disease (CAD) is a leading cause of death world-wide, and most cases have a complex, multifactorial aetiology that includes a substantial heritable component. Identification of new genes involved in CAD may inform pathogenesis and provide new therapeutic targets. The PROCARDIS study recruited 2,658 affected sibling pairs (ASPs) with onset of CAD before age 66 y from four European countries to map susceptibility loci for CAD. ASPs were defined as having CAD phenotype if both had CAD, or myocardial infarction (MI) phenotype if both had a MI. In a first study, involving a genome-wide linkage screen, tentative loci were mapped to Chromosomes 3 and 11 with the CAD phenotype (1,464 ASPs), and to Chromosome 17 with the MI phenotype (739 ASPs). In a second study, these loci were examined with a dense panel of grid-tightening markers in an independent set of families (1,194 CAD and 344 MI ASPs). This replication study showed a significant result on Chromosome 17 (MI phenotype; *p* = 0.009 after adjustment for three independent replication tests). An exclusion analysis suggests that further genes of effect size λ_sib_ > 1.24 are unlikely to exist in these populations of European ancestry. To our knowledge, this is the first genome-wide linkage analysis to map, and replicate, a CAD locus. The region on Chromosome 17 provides a compelling target within which to identify novel genes underlying CAD. Understanding the genetic aetiology of CAD may lead to novel preventative and/or therapeutic strategies.

## Synopsis

Coronary artery disease (CAD), which presents clinically as a heart attack (myocardial infarction) or angina, is a leading cause of death world-wide. The aetiology of CAD is complex with a substantial heritable component. Although there is a huge knowledge-base detailing many aspects of the underlying pathophysiology of CAD, it is likely that undiscovered pathways exist. Positional cloning projects can identify novel susceptibility genes; in the first step genome-wide linkage screens are used to assign loci to specific chromosomes.

The authors have collected 2,036 CAD families from four European countries, in order to maximise the power of detecting genes that confer modest risks. A genome-wide linkage scan identified three promising regions for intensive study; one of the linked regions (Chromosome 17) was confined to families with multiple cases of myocardial infarction and was replicated in a second independent series of families. In addition the linkage scan confirmed a previously identified locus on Chromosome 2. These results demonstrate that novel CAD susceptibility genes are tractable to positional cloning which promises to lead to the identification of new molecular insights into this condition, and hopefully, new treatments.

## Introduction

Coronary artery disease (CAD) is the most common cause of death in industrialised countries, and the prevalence is increasing dramatically in developing countries. The various clinical diagnoses that comprise CAD (e.g. angina, myocardial infarction [MI]) are caused by atherosclerosis, a pervasive degenerative condition in which lipid and fibrous matrix is deposited in arterial vessel walls to form atheromatous plaques. The fibrous caps of some of these plaques sited in coronary arteries may be unstable and rupture. This will release thrombogenic material into the lumen of the vessel leading to coronary thrombosis, vessel occlusion, and subsequent infarction of the myocardium, a critical condition with high mortality. While much is known about CAD, and about some aspects of the underlying pathology of atherosclerosis, new strategies for risk predication and intervention are still needed. There thus remains considerable importance in increasing our understanding of CAD pathophysiology.

Familial clustering of CAD has long been recognised [1], suggesting a genetic contribution to susceptibility for this condition. Studies of rare Mendelian forms of CAD have shown how mutations in genes involved in low-density lipoprotein and high-density lipoprotein metabolism/homeostasis can cause premature CAD, but these mutations are thought to explain a relatively minor fraction of familial CAD. The majority of CAD is believed to be multifactorial with a substantial genetic component. For instance, long-term follow-up of monozygotic and dizygotic twin cohorts have estimated the heritability of fatal coronary events as 57% and 38%, respectively, for men and women [2]. Moreover, there is accumulating evidence from epidemiological studies that the genetic component of CAD risk is only partially explained by classic risk factors that are themselves known to be heritable [3–5]. Even though correction for regression dilution bias could reduce the impact of family history of CAD as an independent risk factor, the implication of these epidemiological studies is that susceptibility genes for CAD might exist that are independent of hypercholesterolemia or arterial hypertension or diabetes. These findings are unsurprising, as studies of the pathophysiology of atherosclerosis have shown the importance of pathways involved in inflammation and innate immunity [6,7]. Consequently, the intriguing possibility exists that identification of susceptibility genes might highlight novel biological pathways involved in atherosclerotic plaque formation and/or rupture, providing opportunities for prevention and therapeutic intervention. On the other hand, susceptibility might be determined principally by novel genes involved in well-known pathways (e.g. cholesterol metabolism). These possibilities have motivated large-scale genetic projects to map and identify susceptibility genes for CAD in an unbiased manner with respect to gene function.

Published genetic linkage studies have implicated several loci for CAD. The first such linkage study was from Finland and showed linkage of premature CAD to loci on Chromosomes 2q21.2–22 and Xq23–26 [8]. Subsequent studies from Germany reported linkage to Chromosome 14q32.2 [9], from Iceland to Chromosome 13q12–13 [10], from the United States in the GENECARD study to Chromosomes 3q13 and 5q31 (with linkage in subgroups to Chromosomes 1q25, 7p14, 19p13) [11] and another U.S. study to Chromosome 1p34–36 [12]. The Icelandic locus was replicated in population-based studies from Iceland and England, with different haplotypes of the *ALOX5AP* gene (encoding 5-lipoxygenase activating protein) associated with CAD in the two countries, and the Icelandic haplotype was also associated with stroke in Iceland and in Scotland [10,13]. The Chromosome 2 linkage identified by the BHF Family Heart Study [14] was also detected in two previous studies [8,12], and provides a promising region for further investigation.

The status of CAD loci which have not been replicated is less clear. Differences in the population studied and/or in the selection criteria used might result in selection for different genetic effects in this heterogeneous disorder. However, the lack of replication might also be expected given that most of these linkage studies had rather low power relative to the anticipated effect size of CAD susceptibility genes. The published linkage screens have ranged in size from 156–1,933 families (~200–2,400 ASPs) and the linkage statistics reported have sometimes been suggestive rather than of firm genome-wide significance, suggesting that some postulated loci may be false positives. None of the published studies have had the statistical power to replicate a specific locus. Rather, the signal-to-noise ratio is such that these studies have had borderline power to detect individual susceptibility genes, and so each will tend by chance to identify a different selection.

The PROCARDIS study was founded as a European collaborative project to assemble a sufficiently large number of families to map the modestly sized susceptibility genes that might plausibly be hypothesised to contribute to CAD risk. We present here the results of the first phase of the study, a linkage study of 2,658 ASPs from 2,036 families with multiple CAD siblings.

## Results

Affected sibling pairs (ASPs) were identified in the families by applying two phenotypic criteria: 1) a broad CAD definition of disease status in the affected sibling(s), (i.e. MI, symptomatic acute coronary syndrome [SACS], chronic stable angina or coronary revascularisation at age < 66 y), with each ASP containing at least one proband with MI or SACS, or 2) a narrow definition of disease based on a clinical history of MI at age < 66 y. Table 1 shows the country of residence of the ASPs in addition to the number of full- and half-siblings confirmed or identified in a RELPAIR [15] relationship analysis. Overall 5% (133/2,658) of the ASPs were reclassified as half-sibling pairs. A further 70 individuals were excluded from analysis as they were apparently unrelated (55 individuals) or possibly distantly related (15 individuals) to the recorded family structure.

**Table 1:**
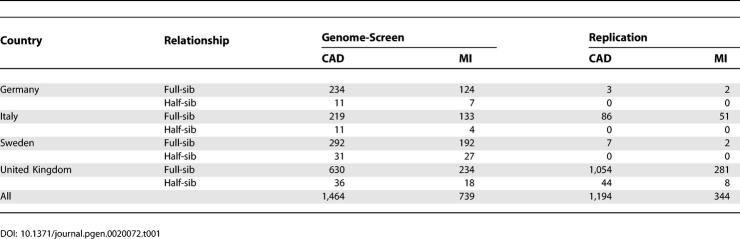
Country of Residence and Degree of Relatedness for Affected Sib-Pairs in the Genome-Screen and Replication Cohorts

**Figure 1:**
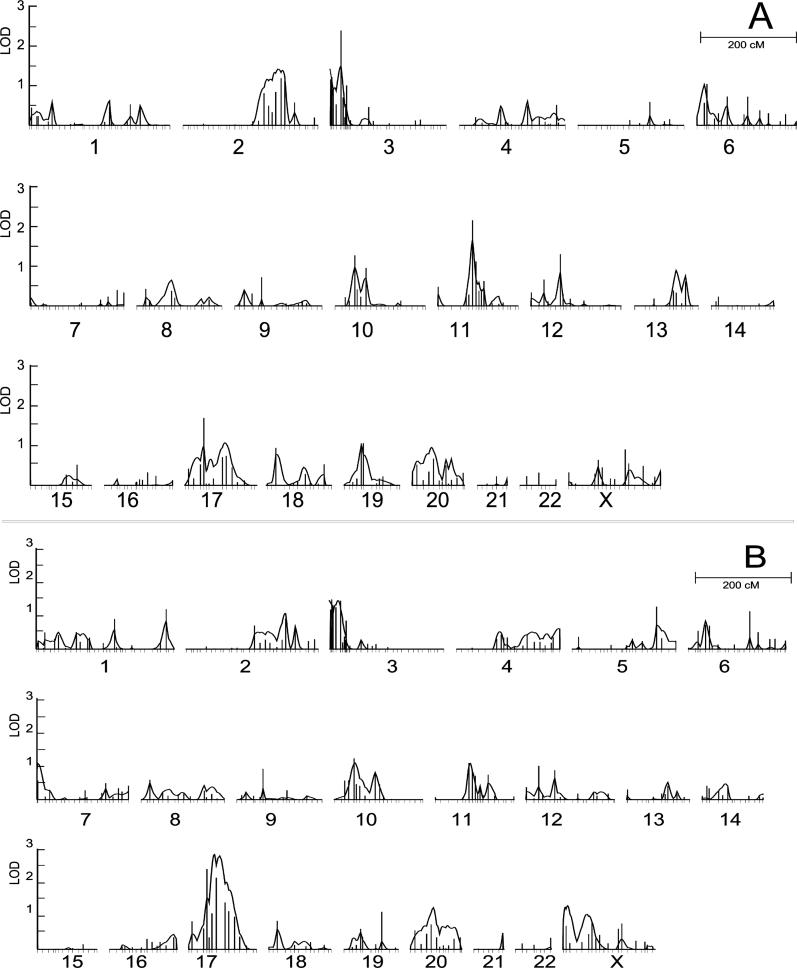
A Genome-Wide Scan of CAD and MI A genome-wide scan of CAD—broad CAD phenotype, 1,464 ASPs (A), and MI—narrow phenotype, 739 ASPs (B). Genetic location (abscissa) is scaled in Kosambi cM. LOD (ordinate) denotes the maximum LOD score statistic. The solid line shows multipoint LODs calculated at regular intervals; the vertical bars show single-point LODs (i.e. calculated at each microsatellite marker). The location of each microsatellite marker is shown by an external tick on the abscissa.

We first undertook an initial genome-screen (Figure 1) for 1,464 CAD ASPs (Figure 1A) and 739 MI ASPs (Figure 1B) genotyped with 446 microsatellite markers. Three regions of tentative linkage were identified by multipoint linkage analysis using the MLSix programme on Chromosomes 3 (CAD phenotype, log of the odds [LOD] = 1.52), Chromosome 11 (CAD phenotype, LOD = 1.66), and Chromosome 17 (MI phenotype, LOD = 2.85). The genome-wide significance of these statistics (i.e. allowing for the multiple significance testing inherent in a genome-screen) was estimated by computer simulation as *p* = 0.81 (CAD, Chromosome 3), *p* = 0.71 (CAD, Chromosome 11) and *p* = 0.08 (MI, Chromosome 17). These results were supported by an alternative multipoint linkage analysis method using the Merlin programme (see Figure S1 for detailed results). For instance, the Merlin analysis showed linkage to the MI phenotype on Chromosome 17 (LOD = 3.19; location = 61 cM; −1 LOD support interval = 41–77 cM).

**Figure 2:**
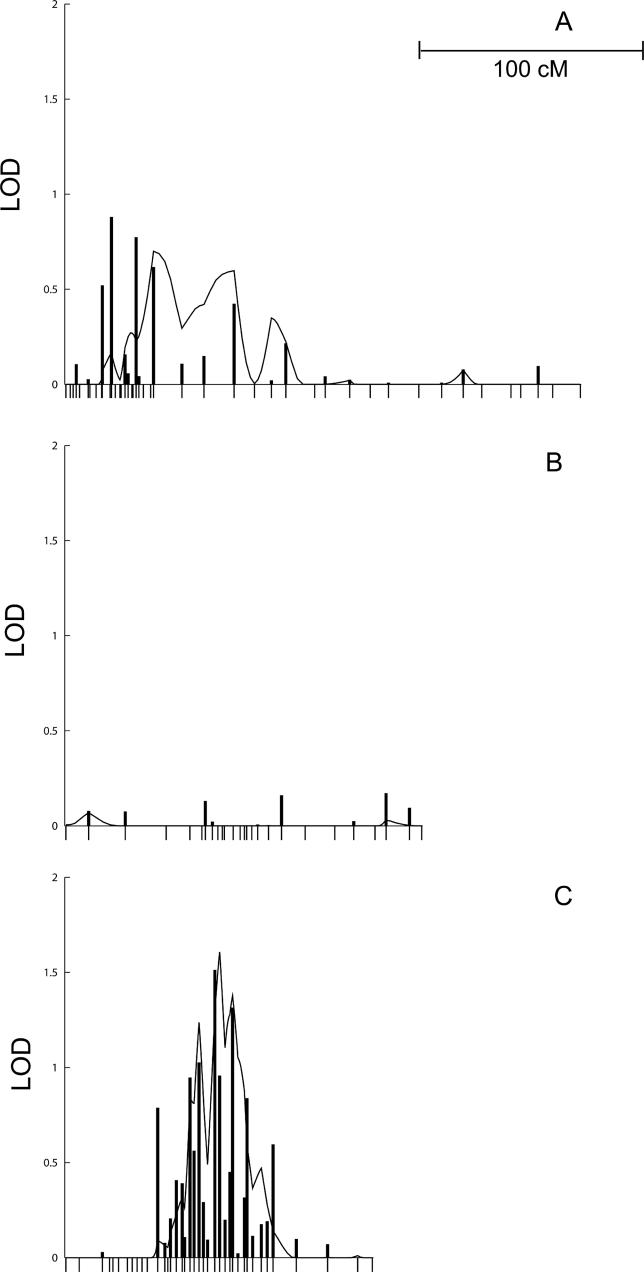
Replication Analysis of Chromosomes 3, 11, and 17 Linkage Peaks Replication analysis, in independent ASPs, of the three Chromosomes (3, 11, and 17) most strongly implicated by the initial genome-wide screen. (A and B) show the results for the broad CAD phenotype and Chromosomes 3 and 11 (1,194 ASPs). (C) shows the results for the narrow MI phenotype and Chromosome 17 (344 ASPs). Genetic location (abscissa) is scaled in Kosambi cM. LOD (ordinate) denotes the MLS statistic. The solid line shows multipoint LODs calculated at regular intervals; the vertical bars show single-point LODs (i.e. calculated at each microsatellite marker). The location of each microsatellite marker is shown by an external tick on the abscissa.

In order to further test the potential importance of the loci identified in the linkage analysis, these regions were tested in an additional sample of 1,194 CAD ASPs and 344 MI ASPs (details shown in Table 1). Table 1 shows that families recruited in the United Kingdom were over-sampled in the replication phase of the project (and German and Swedish families were under-sampled), which simply reflects differences in the timing of ASP recruitment across the four countries. Additional grid-tightening markers (eight markers on Chromosome 3, seven markers on Chromosome 11, and 25 markers on Chromosome 17) were included to maximise the recovery of identity-by-descent information for these regions. Figure 2 summarises the results of these replication experiments. There was insignificant evidence of linkage on Chromosome 3 to the broad CAD phenotype (*p* = 0.056), with a peak LOD = 0.7 mapping 11.9 cM outside the −1 LOD support interval identified in the genome-wide screen (Table 2). There was no evidence for linkage to Chromosome 11 in the broad CAD replication cohort. In contrast, the MI susceptibility locus on Chromosome 17 showed significant evidence for linkage (*p* = 0.003) at location 67 cM (Table 2). After allowance for multiple testing of three independent hypotheses generated in the initial genome-wide screening phase of the experiment, the linkage to Chromosome 17 was significant (*p* = 1 – (1 – 0.003)^3^ = 0.009) [16]. Table 2 shows maximum likelihood estimates and 95% confidence intervals (CI) for the recurrence risk ratio (λ_sib_) associated with the linked region on Chromosome 17 for MI phenotype. The linked region was further investigated in the replication cohort with CAD (1,194 ASPs); there was weak support for linkage between Chromosome 17 and the broad CAD phenotype (*p* = 0.156). Finally, an analysis of the combined data from the genome-wide screen and replication cohorts showed linkage to the MI phenotype on Chromosome 17 (LOD = 2.68; location = 69 cM; −1 LOD support interval = 63–79 cM).

**Table 2:**
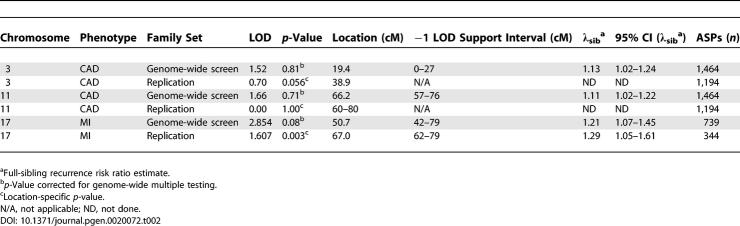
Peak LODs on Chromosomes 3 and 11 (CAD Phenotype) and 17 (MI Phenotype)

**Figure 3:**
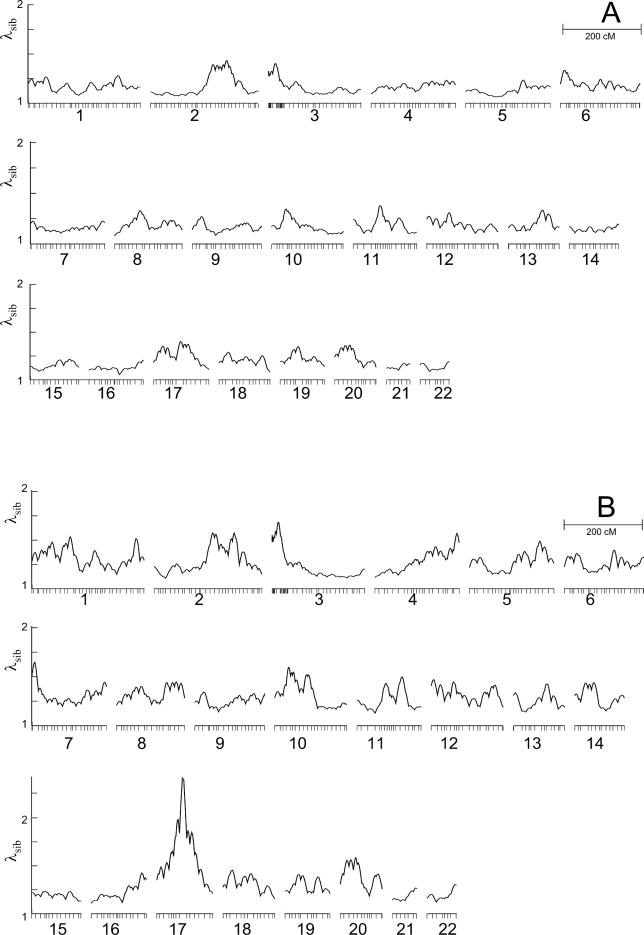
Exclusion Linkage Analysis Exclusion linkage analysis in the initial genome-wide screen set of families. (A) shows the results for the CAD—broad phenotype, and (B) shows the results for the MI—narrow phenotype. Genetic location (abscissa) is scaled in Kosambi cM. The exclusion limit (λ_sib_ associated with a LOD = −2) at each location is plotted. The location of each microsatellite marker is shown by an external tick on the abscissa.

An exclusion analysis (Figure 3) was performed for CAD (Figure 3A) or MI (Figure 3B) ASPs by calculating the largest λ_sib_ at each genetic location associated with a LOD of −2 [17]. Using this strict exclusion criterion, 85% of the autosomal genome was excluded for a locus-specific effect of λ_sib_ = 1.24 (CAD phenotype) and λ_sib_ = 1.42 (MI phenotype).

## Discussion

Susceptibility genes for human complex diseases are believed to confer modest risks and hence genetic linkage to such loci may be difficult to replicate. So our finding that a region of tentative linkage to the MI phenotype identified on Chromosome 17 in a genome-wide screen (*p* = 0.08) was replicated in an independent cohort of families (*p* = 0.009, corrected for multiple comparisons) is important and provides a target for further positional cloning studies.

It is well known that estimates of genetic effect sizes from genome-wide screens are frequently upwardly biased [18] and that more realistic estimates can be obtained in independent replication cohorts. Table 2 provides estimates of the genetic effect size (and their 95% CI) associated with linkage to Chromosome 17 that were obtained by a bootstrap re-sampling procedure. The peak LOD obtained in the genome-screen was at map location 50.7 cM which was associated with an effect λ_sib_ = 1.21; in the replication data, the effect size estimated at the same location was considerably smaller (λ_sib_ = 1.04). The LOD −1 support interval for localising the MI susceptibility gene in the genome-wide screen was broad (42–79 cM) which is typical for complex diseases [19]. The peak LOD in the replication dataset reassuringly falls within this interval at location 67.0 cM (λ_sib_ at this position was 1.29 in the replication cohort).

As the linked region on Chromosome 17 appears to be specific for the MI phenotype, with no evidence for linkage to the broad CAD phenotype in the replication set of ASPs (*p* > 0.156), it is important to consider the possible reasons for this phenomenon. Current knowledge of the pathophysiological processes that lead to CAD indicate that they are highly diverse, and to a large extent not understood in full detail. In addition the CAD disease entity is heterogeneous, consisting of four major diagnostic outcomes: MI, angina, unstable angina, and coronary revascularisation events [20]. There is considerable overlap between these outcomes, yet they differ significantly in their underlying pathophysiology. Coronary artery atherosclerosis forms the basis for all four disease events, yet MI and unstable angina require a further step of rapid deterioration in coronary artery blood-flow. This last step is, in addition to atherosclerotic build-up, determined by a complex interplay between factors such as plaque stability, inflammatory response, platelet function and the coagulation cascade [21]. A modest level of atherosclerosis can be sufficient for some individuals to suffer an acute coronary event, but others with severe coronary artery stenosis never, or only late in life, suffer a clinically detectable MI. An understanding of how this kind of phenotypic, and potentially genotypic, heterogeneity affects our linkage results is important when analyzing large sets of data [22]. Further statistical evaluation, including ordered subset analysis and quantitative-trait linkage analysis, could be usefully applied to promising linked regions in this and other genome screens. Thus, different sets of CAD patients will plausibly differ significantly in their linkage results, a conclusion supported by the fact that few of the loci in the CAD linkage studies published hitherto overlap with each other [8–12,14].

The region of linkage to MI in the present study, flanked by markers D17S921 at 17p11.2 (40 cM; genomic position 14,170,191 in the National Centre for Biotechnology Information 35.1 assembly) and D17S787 at 17q21 (79 cM; position 50,637,083), spans the centromere and includes over 36 megabases of DNA containing over 300 genes of known function and about as many again predicted genes. This interval contains numerous genes that are plausible candidates for involvement in CAD, therefore our future research is focused on refining the region genetically by means of high density SNP coverage, using the PROCARDIS case-control collection and our trio families, and by an analysis of quantitative trait loci (QTL) for CAD intermediate phenotypes measured in the affected sib-pair families. There are published QTLs close to this region that may provide clues. For example, two QTLs for low-density lipoprotein cholesterol map just outside the PROCARDIS region to 17q23.2–25.3 [9] and 17q24.2–25.3 [23]. In familial combined hyperlipidemia pedigrees, a QTL for apoB at 17p11–q21 [24] is within our mapped region, as is a QTL affecting low-density lipoprotein peak particle diameter at 17q21.33 [25]. QTLs for body mass index in two National Heart, Lung & Blood Institute study subsets map to markers D17S947 at 17p12, coincident with a QTL for leptin [26], and D17S2196 at 17p11.2 lie close to the PROCARDIS MI linkage region. Tentative QTLs for smoking behaviour from the Framingham Heart Study map to 17q21.2 and 17q25.3, which is intriguing given the strong links between smoking and CAD risk [27].

A region of linkage using ordered subset analysis in 26 young-onset CAD families has been identified between markers D17S787 and D17S944 (50,637,083–58,790,038 National Centre for Biotechnology Information 35.1 assembly) [14] which adjoins our Chromosome 17 linkage; given the limited resolution of linkage mapping of complex traits [19], these two loci plausibly overlap. A similar deduction may be made for Chromosome 2 (Figure 1A) in which a broad region of linkage (maximum LOD 1.42, CAD phenotype) overlaps distally with earlier findings [8,12,14]. These results encourage detailed examination of these loci in positional cloning and large-scale, gene-association studies.

In conclusion, the results from the PROCARDIS genome-wide linkage analysis are important because they identify a novel replicated locus for MI on Chromosome 17. In addition, our CAD linkage results are consistent with a CAD susceptibility locus mapping to Chromosome 2. The lack of evidence for other linked loci suggests that the heritable component of coronary artery disease and MI may be composed of many genes, with few of them having sufficient effect to permit mapping by genetic linkage even in studies of this size involving thousands of affected sibling pairs.

## Materials and Methods

### Family collections.

Ascertainment criteria for PROCARDIS probands were MI or SACS, on the assumption that the latter represents a similar pathological process according to modified World Health Organisation diagnostic criteria [28,29] before the age of 66 y [30]. Diagnosis of MI required documentation of two or more of: (a) typical ischemic chest pain, pulmonary oedema, syncope or shock; (b) development of pathological Q-waves and/or appearance or disappearance of localized ST-elevation followed by T-wave inversion in two or more standard electrocardiograph leads; (c) increase in concentration of serum enzymes consistent with MI (e.g. creatine kinase more than twice the upper limit of normal). Diagnosis of SACS required documentation of hospitalization for one of the following indications: (a) unstable angina diagnosed by typical ischemic chest pain at rest associated with reversible ST-depression in two or more standard electrocardiograph leads; (b) thrombolysis for suspected MI (as indicated by localized ST-elevation in two or more standard electrocardiograph leads) even without later development of T-wave inversion, Q-waves, or a significant enzyme rise; or (c) emergency revascularization (i.e. during same admission) following presentation with typical ischemic chest pain at rest. Probands completed questionnaires in order to recruit affected siblings with a range of CAD diagnoses at age < 66 y (MI, SACS, chronic stable angina, or intervention for coronary revascularization), who were then invited to participate in the study if their diagnoses were confirmed. Parents and up to four unaffected siblings per family were recruited wherever possible to augment the recovery of linkage phase information. Informative families were recruited in Germany, Italy, Sweden, and the United Kingdom; 99.5% of the study participants reported having a white European ancestry. The protocol was approved by the Ethics Committees of the participating institutions and all participants gave written, informed consent.

### DNA extraction and microsatellite genotyping.

DNA was extracted from 9 ml EDTA-anticoagulated samples of frozen whole blood using Gentra Systems PUREGENE kits (Flowgen, Lichfield, United Kingdom) according to the manufacturer's instructions, and its concentration determined fluorimetrically using SYBR^®^ Green. DNA was plated and genotyped for the ABI MD10 (Applied Biosystems, Warrington, United Kingdom) set of microsatellite markers using ABI 3700 capillary sequencing equipment [31]. All microsatellite genotyping was carried out at Oxagen Limited (Abingdon, Oxfordshire, United Kingdom). Genotyping was carried out blind of pedigree structure and disease status. Genotypes were called using the automated functions within the ABI Genotyper software and manually checked independently by two operators. All discrepancies were reviewed and re-genotyped if no consensus call was made. Following a relationship analysis (see below), genotypes were checked for Mendelian inconsistencies [32]. After a further round of re-genotyping, any remaining inconsistent genotypes were flagged as “missing” and removed from any subsequent analysis. The accuracy of genotyping can be estimated in 19 pairs of siblings that, after relationship analysis, were proven to be monozygotic twin-pairs. 5,741/5,759 (99.7%) of their genotypes were concordant. For these 19 monozygotic twins, no genotypes were flagged as “missing” during QC.

Additional grid-tightening markers were genotyped for the linked regions on Chromosomes 3 (eight markers), 11 (seven markers), and 17 (25 markers); details are included in Table S1. A total of 15 markers (MD10 + grid-tightening markers) were completely excluded from further analysis following QC analysis.

### Statistical analysis.

Genetic relationships between cognate relatives were confirmed in an analysis using the RELPAIR program [15] with all genotyped microsatellite markers. If sibling pairs were found to be consistent with being related at the half-sib level, their family structures were revised and included in subsequent analyses. Individuals found to be unrelated to their recorded family were excluded from all subsequent analyses. Any remaining inconsistent genotypes were subsequently marked as missing. Two further grid-tightening markers were eliminated as they showed excessive non-Mendelian inheritance and Hardy-Weinberg disequilibrium. The genetic map for all of the genome-screen and majority of the grid-tightening markers was extracted from the Rutgers sex-averaged map [33]. The Rutgers map is an integrated genetic map in which physical sequence data was used to establish marker order; genetic map distances were estimated by maximum likelihood methods using meiotic mapping data from Centre d'Etude Polymorphism Humaine and DeCode genotype databases. The locations of five grid-tightening markers absent from the Rutgers map were linearly interpolated with respect to flanking markers that were included in the Rutgers map.

Non-parametric linkage analysis was undertaken using the computer program MLSix, to compute single-point and multi-point maximum LOD score (MLS) statistics [34,35] for affected full and half sib-pairs [17]. For autosomal loci, two parameters were included to model additive and dominance effects on susceptibility, and LODs were maximized under the “possible triangle” constraints [34]. For X-linked loci, LODs were maximised under analogous linear constraints [36] for same or mixed-sex full-sibs and informative half-sibs (maternal half-brothers and half-brother-sisters and half-sisters). ASPs drawn from affected sibships of size ≥ 2 were equally weighted in the MLS calculations. All ASPs include at least one affected sib, complying with the PROCARDIS proband definition (MI or SACS at age < 66 y). Identity-by-descent vectors for the full and half-sib pairs for each sibship were calculated by the Merlin program [37], using the “minx” version of the program for X-linked data. Marker allele frequencies were estimated by gene counting pooling information across the four countries and across genome-screen and replication cohorts where available. In regions of linkage, the peak LOD is associated with a measure (recurrence risk ratio in full-siblings −λ_sib_) of the genetic effect size for this susceptibility gene; 95% CI for this λ_sib_ estimate were calculated using a bootstrapping technique with 1,000 replicates [38]. Exclusion analysis was performed using MLSix; the maximization function was modified to estimate the λ_sib_ associated with a MLS = −2 (a strict exclusion threshold) at each genetic location. This analysis is able to show in regions that show little or no evidence of linkage, that there is sufficient identity-by-descent information to confidently exclude susceptibility genes of modest effect sizes. The statistical significance of the MLS statistics were established by a computer simulation which modeled the family structures (including the patterns of missing parental genotypes and the small proportion of families with more than two affected sibs), marker density and informativity [17]; 1,000 replicates of a complete genome-wide screen were simulated. To interpret the significance of the MLS statistic in the genome-wide screen (i.e. allow for multiple testing), the number of MLS statistics greater than desired threshold across all chromosomes was recorded. To interpret the significance of the MLS statistic in the replication experiment, the number of MLS greater than desired threshold at a one location (i.e. one test at one specific location) was recorded.

The authors had full access to the data and take responsibility for its integrity. All authors have read and agree to the manuscript as written.

## Supporting Information

Figure S1 A Genome-Wide Scan of CAD and MI A genome-wide scan of CAD—broad CAD phenotype, 1,464 ASPs (A) and MI—narrow phenotype, 739 ASPs (B). Genetic location (abscissa) is scaled in Kosambi M. The ordinate denotes the Kong & Cox LOD score [39] calculated using the Merlin computer programme [37]. The solid line shows multipoint LOD scores calculated at regular intervals. For details of the Merlin programme see http://www.sph.umich.edu/csg/abecasis/Merlin/index.html
(586 KB TIF)

Table S1 40 Additional Grid-Tightening Markers on Chromosomes 3, 11, and 17 (62 KB DOC)

## Abbreviations

ASP: affected sibling pair
CAD: coronary artery disease
CEPH: Centre d'Etude Polymorphism Humaine
cM: centiMorgan
LOD: log of the odds
MI: myocardial infarction
MLS: maximum LOD score
QTL: quantitative trait locus
SACS: symptomatic acute coronary syndrome
