# Supplementary material for: Genome-Wide Mapping of Susceptibility to Coronary Artery Disease Identifies a Novel Replicated Locus on Chromosome 17
Source: PLoS Genet. 2006 May 19;2(5):e72. doi: 10.1371/journal.pgen.0020072 (PMC1463045; doi:10.1371/journal.pgen.0020072)
Supplement: Supplementary file 1 [file pgen.0020072.st001.doc]

**Supplementary Table 1**:

**Table of 40 additional grid-tightening markers on chromosomes 3, 11 and 17**. If a marker has not been assigned a “D number”, a pair of forward and reverse primers is listed.

| **Chromosome** | **Marker** | **Forward Primer** | **Reverse Primer** |
| --- | --- | --- | --- |
| 11 | D11S1321 |  |  |
| 11 | D11S1361 |  |  |
| 11 | D11S1993 |  |  |
| 11 | D11S4076 |  |  |
| 11 | D11S4136 |  |  |
| 11 | D11S4162 |  |  |
| 11 | D11S4205 |  |  |
| 17 | D17S1166 |  |  |
| 17 | D17S1838 |  |  |
| 17 | D17S1824 |  |  |
| 17 | D17S1815 |  |  |
| 17 | D17S1805 |  |  |
| 17 | D17S1795 |  |  |
| 17 | D17S1791 |  |  |
| 17 | D17S1787 |  |  |
| 17 | D17S2194 |  |  |
| 17 | D17S1293 |  |  |
| 17 | D17S669 |  |  |
| 17 | D17S1356 |  |  |
| 17 | D17S936 |  |  |
| 17 | D17S969 |  |  |
| 17 | D17S960 |  |  |
| 17 | D17S1861 |  |  |
| 17 | D17S945 |  |  |
| 17 | D17S933 |  |  |
| 17 | D17S927 |  |  |
| 17 | D17S918 |  |  |
| 17 | D17S838 |  |  |
| 17 | D17S805 |  |  |
| 17 | D17S790 |  |  |
| 17 | D17S731 |  |  |
| 17 | D17S957 |  |  |
| 3 |  | GAGATAATCTGAGGCATGGAGA | GAGCTTTAAAGTCCTATACCAACA |
| 3 |  | GCTTCCAAAATGCCTCCA | GTTTCCTGGTTGGCTGTTTTA |
| 3 | D3S2397 |  |  |
| 3 | D3S3591 |  |  |
| 3 | D3S3630 |  |  |
| 3 | D3S3691 |  |  |
| 3 | D3S3706 |  |  |
| 3 | D3S4538 |  |  |
